# Functional Imaging for Therapeutic Assessment and Minimal Residual Disease Detection in Multiple Myeloma

**DOI:** 10.3390/ijms21155406

**Published:** 2020-07-29

**Authors:** Bastien Jamet, Elena Zamagni, Cristina Nanni, Clément Bailly, Thomas Carlier, Cyrille Touzeau, Anne-Victoire Michaud, Philippe Moreau, Caroline Bodet-Milin, Françoise Kraeber-Bodere

**Affiliations:** 1 Nuclear Medicine/Hematology Department, Nantes University Hospital, F-44000 Nantes, France; bastien.jamet@chu-nantes.fr (B.J.); clement.bailly@chu-nantes.fr (C.B.); thomas.carlier@chu-nantes.fr (T.C.); cyrille.touzeau@chu-nantes.fr (C.T.); annevictoire.michaud@chu-nantes.fr (A.-V.M.); philippe.moreau@chu-nantes.fr (P.M.); caroline.milin@chu-nantes.fr (C.B.-M.); 2 Seràgnoli Institute of Hematology, Bologna University School of Medicine, 40126 Bologna, Italy; e.zamagni@unibo.it; 3 Nuclear Medicine Department, Azienda Ospedaliero-Universitaria di Bologna, 40138 Bologna, Italy; cristina.nanni@aosp.bo.it; 4 CHU de Nantes, CNRS, Inserm, CRCINA, Université de Nantes, F-44000 Nantes, France; 5 Nuclear Medicine Department, ICO René Gauducheau, F-44800 Saint-Herblain, France

**Keywords:** MM, imaging, therapeutic assessment, FDG-PET/CT, prognostic value, MRD

## Abstract

Serum markers and bone marrow examination are commonly used for monitoring therapy response in multiple myeloma (MM), but this fails to identify minimal residual disease (MRD), which frequently persists after therapy even in complete response patients, and extra-medullary disease escape. Positron emission tomography with computed tomography using 18F-deoxyglucose (FDG-PET/CT) is the reference imaging technique for therapeutic assessment and MRD detection in MM. To date, all large prospective cohort studies of transplant-eligible newly diagnosed MM patients have shown a strong and independent pejorative prognostic impact of not obtaining complete metabolic response by FDG-PET/CT after therapy, especially before maintenance. The FDG-PET/CT and MRD (evaluated by flow cytometry or next-generation sequencing at 10^−5^ and 10^−6^ levels, respectively) results are complementary for MRD detection outside and inside the bone marrow. For patients with at least a complete response, to reach double negativity (FDG-PET/CT and MRD) is a predictive surrogate for patient outcome. Homogenization of FDG-PET/CT interpretation after therapy, especially clarification of complete metabolic response definition, is currently underway. FDG-PET/CT does not allow MRD to be evaluated when it is negative at initial workup of symptomatic MM. New PET tracers such as CXCR4 ligands have shown high diagnostic value and could replace FDG in this setting. New sensitive functional magnetic resonance imaging (MRI) techniques such as diffusion-weighted MRI appear to be complementary to FDG-PET/CT for imaging MRD detection. The goal of this review is to examine the feasibility of functional imaging, especially FDG-PET/CT, for therapeutic assessment and MRD detection in MM.

## 1. Introduction

Multiple myeloma (MM) is a plasma cell malignancy characterized by a clonal population of bone marrow plasma cells that secrete a monoclonal paraprotein or an immunoglobulin free light chain. Whilst the detection of MM can result from a broad range of indications, including anemia, hypercalcemia, and renal failure, the most common symptom is lytic bone disease owing to an over-activation of osteoclasts, and occurs in over 80% of patients [1]. Imaging plays a crucial role in the detection of premature lytic bone lesions and modern morphological or hybrid imaging techniques have gradually replaced conventional skeletal surveys which should no longer be performed except when these innovative imaging techniques are not available. It is now recommended [2] to perform whole-body computed tomography (WBCT) as stand-alone CT, or as part of a positron emission tomography with CT using an 18F-deoxyglucose (FDG-PET/CT) protocol in the first instance at symptomatic MM diagnosis.

Novel and effective agent-based therapies have led to significant improvements in the treatment of MM patients, with many achieving a profound biological response. This is a crucial goal for transplant-eligible newly diagnosed MM patients, because of its major prognostic impact. Whilst serum markers and bone marrow examination is routinely used for monitoring response to therapy in MM [3], it fails to identify minimal residual disease (MRD), which frequently persists after therapy, even in complete response patients, and extra-medullary disease escape. Modern biological diagnostic tools, such as multiparametric flow cytometry, next generation sequencing and functional imaging techniques such as FDG-PET/CT, allow the detection of increasingly lower levels of disease. This has led to a strengthening of the definition of complete response, and to the introduction of new concepts in MRD detection inside the bone marrow as well as imaging plus MRD in patients with complete response [4].

MM induces a high spatial genomic heterogeneity, resulting in the cohabitation of distinct disease clones within a patient that exhibit various genomic profiles in the bone marrow and in bone focal lesions [5]. Moreover, this genomic heterogeneity increases with the size of the focal bone lesions depicted by FDG-PET/CT. Detection of MRD within and outside of the bone marrow in patients with biological complete response is therefore crucial to ensure the full eradication of tumor clones in all compartments, and is clearly linked to long-term outcome. The goal of this review is to highlight the potential of functional imaging, especially FDG-PET/CT, for therapeutic assessment and MRD detection in MM.

## 2. FDG-PET/CT for Therapeutic Assessment of Multiple Myeloma

FDG-PET/CT is the gold-standard imaging technique for therapeutic assessment in MM [2,6]. FDG-PET/CT uses a radiolabeled glucose analog tracer called FDG [7]. After intravenous injection, FDG uptake into metabolically active cells occurs via the classic GLUT family of membrane receptors. Similar to normal glucose, it is then phosphorylated by hexokinase, but it becomes trapped in the cell because the C-2 hydroxyl group has been substituted by F-18, and the molecule cannot be fully metabolized to pyruvate. FDG then allows an assessment of glucose uptake, phosphorylation and metabolic activity [8].

**Figure 1:**
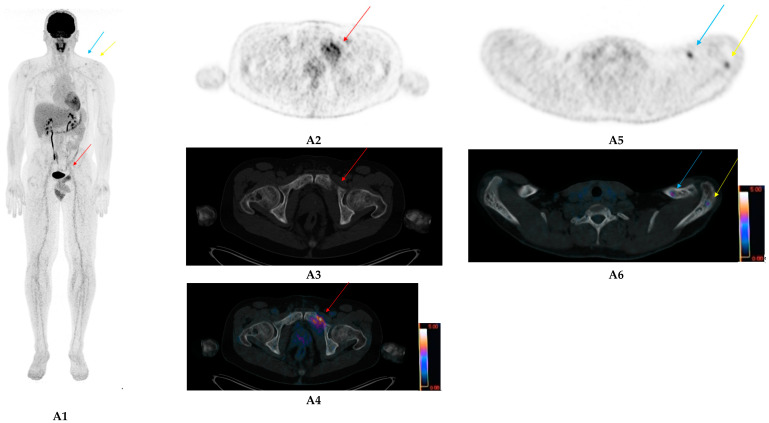

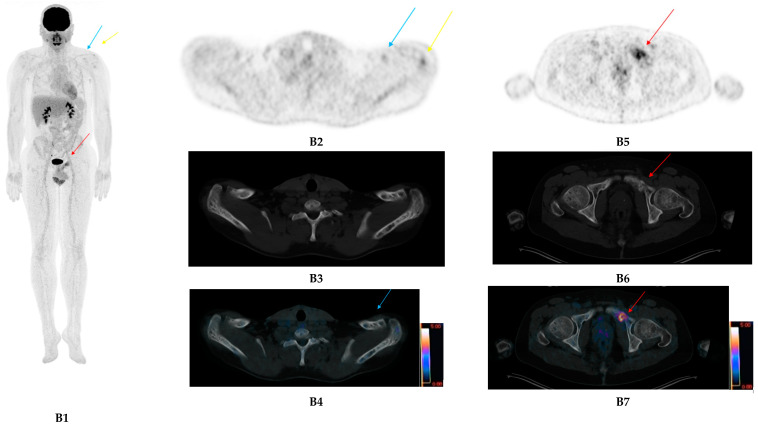
IgGk multiple myeloma (MM) patient at first relapse after autologous stem cell transplantation. Positron emission tomography with computed tomography using 18F-deoxyglucose (FDG-PET/CT) images before therapy (**A**) show several focal bone lesions (FLs) in maximum intensity projection (MIP) images (**A1**), especially the left pelvic FL (Red arrow; (**A2**) PET images; (**A3**): computed tomography (CT) images; (**A4**) fused images, maximum standardized uptake value (SUV_max_: 9)) and left collarbone (Blue arrow)/acromion FLs (Yellow arrow) in axial slices (**A5**/**A6**). Note the numerous osteolytic bone lesions without corresponding increased FDG uptake in the CT images as this patient had been pretreated, and morphological abnormalities may persist a long time after. FDG-PET/CT images after therapy (**B**) shows overall decrease in FL uptake in MIP images (**B1**). Whilst the left collarbone(Blue arrow)/acromion FL (Yellow arrow) residual uptake (**B1**/**B2**/**B3**/**B4**) is lower than the hepatic background (Deauville 3), the left pelvic FL (Red arrow) residual uptake (**B1**/**B5**/**B6**/**B7**) has decreased but is still higher than the hepatic background (Deauville 4, SUV_max_: 6) revealing a partial metabolic response.

The great strength of FDG-PET/CT after therapy for MM is that it allows the detection of residual active clonal plasma cells within residual lytic bone lesions. These commonly persist for a long time after the start of therapy. As shown in Figure 1, CT alone is not a suitable imaging technique for monitoring response to therapy in (all?) bone lesions.

Conversely, sclerotic changes in lytic lesions such as traditional callus formation around rib lesions or sclerotic rim development around axial and peripheral skeleton lesions can lead to the misinterpretation of FDG-PET/CT images and result in false-positive post-therapy images.

Since the seminal study published by Bartel et al. in 2009 [9], all large-scale prospective studies of transplant-eligible newly diagnosed MM cohorts have supported the powerful prognostic value of FDG-PET/CT examinations after first-line therapy. These have revealed that a return to normal in all focal bone lesions’ uptake before the autologous stem cell transplantation process (then after chemotherapy induction cycles) is highly correlated with longer progression-free and overall survival, and can be useful as a faithful predictive surrogate for patient outcome which is primordial at age of targeted and risk-based therapies. In 2013, the same team [10] examined a larger series of 302 patients treated using an intensive protocol consisting of two consecutive autologous stem cell transplantations. The gene expression profiles of 277 of these patients were also examined. Persistence of significant uptake of three focal bone lesions on FDG-PET/CT imaging carried out seven days after the start of induction was significantly correlated with lower progression-free and overall survival in the whole cohort, and additionally in the sub-group of patients harboring high-risk gene expression profiles. These findings imply that FDG-PET/CT imaging could be regarded as a safe instrument for very premature therapeutic adjustment. Finally, in 2018, the identical team corroborated these findings for a wider cohort including more than 500 patients encompassed in their TT4-TT6 clinical trials. This study [11] aimed to confirm the prognostic significance of the suppression of focal bone lesion uptake at multiple time points after therapy initiation. All patients underwent serial FDG-PET/CT imaging: at baseline, seven days after the start of therapy, after induction therapy was completed, after autologous stem cell transplantation and finally before maintenance therapy. Patients with no residual uptake in focal bone lesions (compared to background bone marrow signal) seven days after the initiating treatment showed progression-free and overall survival values similar to patients without focal bone lesions at diagnosis. At later time points, patients who reached continuous total suppression of focal bone lesions uptake had statistically equivalent progression-free and overall survival values compared to patients without metabolically vibrant focal bone lesions at baseline. These important conclusions emphasized the importance of full suppression of focal bone lesions as a major therapeutic target for newly diagnosed MM patients with focal bone lesions.

In 2011, Zamagni et al. explored the prognostic relevance of FDG-PET/CT after thalidomide-dexamethasone induction therapy and double autologous stem cell transplantation in 192 newly diagnosed MM patients [12]. Firstly, they confirmed the relevance of FDG-PET/CT imaging performed after induction therapy because patients harboring a maximum standardized uptake value (SUV_max_, traducing intensity of carbohydrate metabolism) of residual focal bone lesions > 4.2 had a significantly lower progression-free survival. Secondly, patients were assessed by FDG-PET/CT imaging 3 months after autologous stem cell transplantation. Complete metabolic response, defined similarly to Bartel et al. as the complete absence of residual uptake of the initial targets, was reached for 65% of patients. Progression-free and overall survival of these patients at 4 years was significantly higher than for the PET-positive patients (47% vs. 32% and 79% vs. 66%, respectively). Moreover, 23% of all patients who achieved complete biological response according to the IMWG’s regular guidelines harbored an FDG-PET/CT-positive lesion and were associated with poorer outcome, meaning residual FDG uptake after therapy could detect MRD. Multivariate analysis of all statistically significant prognostic variables by univariate analysis confirmed that FDG-PET/CT status after autologous stem cell transplantation (before maintenance therapy) was one of the most significant independent predictors of progression-free survival. Afterwards, in 2015, the same group reproduced these results in a cohort of 282 symptomatic MM patients initially treated between 2002 and 2012 [13]. FDG-PET/CT was carried out at baseline, before maintenance therapy and at relapse. The strongest baseline variables independently correlated with progression-free and overall survival by multivariate analysis were an initial FDG-PET/CT-derived feature (SUV_max_ of focal bone lesions >4.2), an international staging system > 2 and a failure to reach complete biological response. These three features were then combined to build a prognostic scoring system. After therapy, FDG-PET/CT negativity (defined above) was reached in 70% of patients, while only 53% percent of patients reached a conventional biological complete response. Once again, FDG-PET/CT negativity after therapy (before maintenance) clearly positively influenced progression-free and overall survival of patients. FDG-PET/CT negativity was independently correlated with extended progression-free and overall survival, even in the biological complete response patient sub-group. Of the sixty-three percent of patients who relapsed or progressed after first-line therapy, bone progression was identified exclusively by systematic FDG-PET/CT in 12% of them. Residual focal bone lesions uptake of SUV_max_ >4.2 after first-line therapy was independently correlated with exclusive FDG-PET/CT progression.

More recently, the prospective multicenter French IMAJEM trial [14] aimed to compare prognostic values of magnetic resonance imaging (MRI) and FDG-PET/CT. In this study, 134 patients were randomized to receive combined lenalidomide, bortezomib, and dexamethasone (RVD) with or without autologous stem-cell transplantation, followed by lenalidomide maintenance. FDG-PET/CT and MRI were carried out at diagnosis, after three cycles of RVD and before maintenance therapy. A normal MRI result after three cycles of RVD and before maintenance was not predictive of progression-free and overall survival owing to a significant number of false-positive patients. FDG-PET/CT normalization after three cycles of RVD was observed for 32% of the patients with positive initial imaging, and progression-free survival was significantly enhanced for these patients (30-months progression-free survival, 78.7% vs. 56.8%). FDG-PET/CT normalization before maintenance therapy was observed for 62% of the patients with initial positive imaging, and was strongly associated with better progression-free and overall survival compared to patients without normalization (24-months progression-free survival: 72 % vs. 57 % and 24-months overall survival: 94 % vs. 73 %, respectively). Interestingly, FDG-PET/CT complete metabolic response in this trial was defined as the residual uptake of initial focal bone lesions, bone marrow, and extra-medullary potential disease targets compared to the background liver uptake. Using multivariate analysis, a return to normal of pre-maintenance FDG-PET/CT was found to be independently correlated with longer progression-free survival, such as no extramedullary disease at baseline and the depth of biological conventional response after three cycles of induction therapy (very good partial biological response or better) suggesting pre-maintenance therapy is the best time to perform FDG-PET/CT for therapeutic assessment.

A second analysis of the IMAJEM cohort that only considered patients with baseline FDG-avid focal bone lesions and/or bone marrow involvement (determined by at least Deauville 4-point scale disease uptake) corroborated the powerful prognostic value of FDG-PET/CT results after three cycles of induction therapy [15]. Indeed, by multivariate analysis, a decrease in the SUV_max_ of the initial target (ΔSUV_max_) was the strongest independent prognostic factor regarding to hazard ratio, which was the more discriminative, especially as compared to conventional biological response. Patients with a reduction in uptake of more than 25% after three cycles of induction therapy had a significantly improved progression-free survival.

Using a smaller prospective cohort of 107 MM patients, Nanni and colleagues also analyzed the prognostic impact of FDG-PET/CT after therapy [16]. Patients were prospectively enrolled and underwent FDG-PET/CT imaging at baseline, three months after autologous stem cell transplantation and then during follow-up every six to twelve months. Forty-seven of the 107 patients relapsed during follow-up. Twenty-two patients had a negative post-therapy FDG-PET/CT, whereas 15 had a positive post-therapy FDG-PET/CT. There was a significant difference between the median response times of the two groups; 27 months for the PET negative patients and only 18 months for the PET positive patients. Not surprisingly, among patients with a positive PET after therapy, the higher the SUV_max_, the shorter the time to relapse. Among patients who did not relapse during follow-up, 27 had a negative post-therapy PET, whereas 13 had a positive post-therapy PET. None of the patients who did not relapse had an SUV_max_ progressive increase during the follow-up.

The open issue concerning FDG-PET/CT interpretation after therapy, notably before maintenance, is to find out the uptake cut-off of residual lesions corresponding to complete metabolic response definition. Indeed, in the trials summarized above, complete metabolic response was not similarly defined (Table 1), and a homogenization has to be done in order to permit meaningful comparisons. This quest for better residual uptake cut-off corresponding to complete metabolic response and MRD definition is currently in progress. The initial findings of a joint analysis of two independent prospective randomized phase III European trials (Italian and French) were presented by Zamagni and colleagues at the 2018 annual meeting of the American Society of Hematology [17]. From this combined analysis of 236 patients, the strongest independent predictor of prolonged progression-free and overall survival was a Deauville score < 4 before maintenance therapy, and was determined using focal bone lesions, bone marrow, and extramedullary disease compared to hepatic background uptake. The final results of this joint analysis are expected to be published soon.

**Figure 2:**
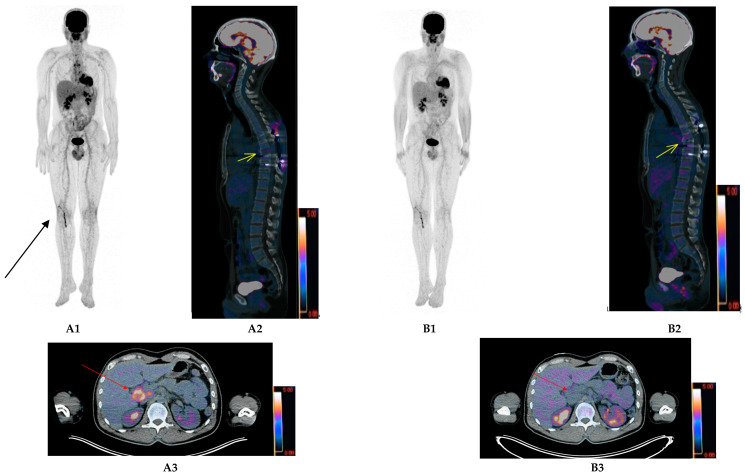
Thirty-nine-year-old patient with a IgAk MM revealed by a thoracic spinal cord compression. FDG-PET/CT images performed at diagnosis (**A1**: Maximum intensity projection (MIP) images) show a T7 vertebral body destruction with corresponding increased uptake in the sagittal fused slice (Yellow arrow **A2**) and a highly metabolically active liver hilary lymph node (Red arrow, **A3** axial fused slice) suspect of extra-medullary disease (EMD). Of note, a recent right knee trans-arthroscopic reconstruction of the anterior cruciate ligament (ACL) by the Kenneth-Jones technique explaining the corresponding uptake (Black arrow in **A1** image). FDG-PET/CT images performed after autologous stem cell transplantation (before maintenance therapy) show complete metabolic response (**B1**) with no significant residual uptake (<mediastinum blood pool, Deauville 2) of T7 vertebral body in sagittal fused slice (Yellow arrow, **B2**) and liver hilary lymph node in axial fused slice (Red arrow, **B3**).

Finally, in the large international CASSIOPET study, a companion study of CASSIOPEIA [18], pre-maintenance complete metabolic response was defined as the residual tumor uptake < mediastinum blood pool background (Figure 2) and uncertain complete metabolic response as the residual tumor uptake <liver background. The preliminary results [19] show that of 184 patients with pre-maintenance PET results (101 treated with daratumumab+bortezomib/thalidomide/dexamethasone (D-VTd) and 83 by Vtd only), 118 (64.1%) achieved CMR, 47 (25.5%) uCR, 17 (9.2%) partial response (PR) and 2 (1.1%) stable disease (SD). D-VTd prolonged progression-free survival in the patients with PET pre-maintenance negativity. One hundred and two and 43 patients were MRD- (evaluated by MFC or NGS) and PET/CT-negative at MRD levels of 10^−5^ and 10^−6^, respectively. Concordance of the PET/CT and MRD results in ≥CR patients (PET-CR/MRD) identified 60 and 28 PET-CR-negative patients at 10^−5^ and 10^−6^ levels, respectively. Pre-maintenance double negativity rates for PET/CT and MRD were higher in D-VTd-treated patients compared to the Vtd patients (66.7% and 47.5% at 10^−5^ and 39.4% and 25.0% at 10^−6^, respectively). Because PET and MRD results are complementary, PET-CR/MRD negativity concordance may provide insight into using both methods as a predictive surrogate for patient outcome.

## 3. A Novel PET Tracer (CXCR4) for Therapeutic Assessment of Multiple Myeloma

Rasche et al. recently published a study that examined the FDG-PET/CT false-negative rate within a cohort of 227 newly diagnosed MM patients, and identified the tumor-intrinsic parameters associated with this pattern [20]. Whilst 11% of patients were FDG-PET/CT negative, this was not linked to the degree of bone marrow plasma cell infiltration or plasma cell proliferation. They then showed a statistically significant decrease in hexokinase-2 expression in this subset, an enzyme that catalyzes the first phosphorylation step of glycolysis, therefore providing a mechanistic reason. More recently, Abe et al. [21] investigated the prognostic impact of low hexokinase-2 expression associated with false-negative FDG-PET in MM patients. Ninety patients with newly diagnosed MM were enrolled in this retrospective study and the authors confirmed that an FDG-PET negativity rate of 12% was associated with low expression of hexokinase 2. Moreover, progression-free survival of false-negative FDG-PET patients was significantly improved compared to FDG-PET positive patients. In addition, progression-free survival rates were comparable between false-negative FDG-PET patients and those without recognized high-risk FDG-PET features.

Thus, FDG-PET/CT is not suitable for MM therapeutic assessment and MRD detection in this sub-group of baseline false-negative FDG-PET patients.

The growth in molecular imaging developments is highly relevant for MM imaging. Novel PET tracers aiming to target different metabolic pathways or plasma cell receptors have demonstrated interesting preliminarily results for disease detection and could be used for MRD detection, especially in FDG-PET/CT negative patients. These include methionine, which is an amino-acid PET tracer, lipid tracers such as choline or acetate, and other promising immuno-PET targets such as CD138 and CD38. Because we recently reviewed several of these novel tracers [22], and because data published about MM therapeutic assessment concern only ^68^Ga-Pentixafor ligand targeting CXCR4 up until now, here we restrict our review to the potential interest of this CXCR4 tracer for therapeutic assessment and MRD detection in MM.

CXCR4 is a G-protein-coupled chemokine receptor family member [23]. CXCL12 (stromal cell-derived factor-1) binds to CXCR4 and triggers different downstream signaling pathways. This results in a variety of responses central to tumor growth and progression including chemotaxis, cell survival and/or proliferation, and gene transcription. The CXCL12/CXCR4 pathway is also implicated in cell migration, homing of hematopoietic stem cells to the bone marrow, angiogenesis and cell proliferation. CXCR4 is overexpressed in a large number of different tumors, including MM [24], and CXCR4 expression correlates with MM disease progression and outcome [25]. CXCR4-directed PET imaging has predominantly been used to assess MM, and around two thirds of these patients show elevated receptor expression on the monoclonal plasma cells’ surface. ^68^Ga-Pentixafor, a labelled peptide with high affinity for CXCR4 and an excellent signal-to-noise ratio in CXCR4-expressing patients is a promising PET ligand [26,27,28], especially because it is possible to theranostically target MM using this reagent. Indeed, this same PET ligand can also be labelled with therapeutic ß-emitters such as ^177^lutetium or ^90^yttrium. Initial CXCR4-directed endo-radiotherapy with ^177^Lu- and ^90^Y-labeled pentixather [29] for progressive refractory MM with massive bone marrow and extra-medullary disease showed promising results, including good treatment tolerance and improved preliminary response rates.

There are still discrepancies between FDG and CXCR4 tracers with respect to disease detection. FDG remains more sensitive than CXCR4 for a large proportion of cases. Indeed, receptor expression on the monoclonal plasma cell surface appears to be an active process that could be deeply impacted by a preceding or concomitant chemotherapy [30], and further studies to clarify this are required. Moreover, no prognostic data about CXCR4-directed PET imaging results before or after therapy have been reported yet.

## 4. New Functional MRI Approaches for Therapeutic Assessment of Multiple Myeloma

Conventional morphological MRI imaging is not a reliable tool for the therapeutic assessment of MM because focal bone abnormalities can persist a long time after the start of therapy without any vital cells, resulting in a high false-positive imaging rate during follow-up [12]. In the attempt to improve monitoring responses to therapy by MRI, new functional MRI sequences have been developed and have shown interesting results. Diffusion-weighted magnetic resonance imaging (DW-MRI) produces images showing discrepancies of water movements in tissues in extracellular spaces, and therefore directly reflects cell density. Imaging is repeated with at least two b values (diffusion weighting), which permits the automated calculation of the apparent diffusion coefficient (ADC) for each voxel in the image and the production of a quantitative map. Tumors therefore appear as areas where water diffusion is restricted (high signal on source diffusion image and low value on ADC map). In MM, it has been demonstrated that the optimal b value for normal and diseased bone marrow contrast discrimination is around 1400 smm^−2^ [31]. However, reaching such a high b value causes technical issues, and a b value of 900 smm^−2^ is commonly chosen. DW-MRI produces high quality image contrast between normal and diseased bone marrow, especially compared to conventional morphological sequences [32,33,34]. Because the response to therapy induces a decrease in cellularity, the high b value signal is reduced, and the ADC values are increased [35,36,37].

In MM, ADC percent changes seem to be significantly higher in good responder patients (biological complete response or very good partial response) compared to patients without a profound response (partial response, minimal response, stable or progressive disease) [38]. Changes in ADC after therapy are now included in the recent MY-RADS MRI guidelines [39]. Whilst these values help to define the response assessment category, ADC cut-off values should be considered with caution, because ADC values are influenced by a lot of different parameters [40].

The potential of DW-MRI for MRD detection in MM after therapy has also been investigated [41] in a study of 168 patients with biological complete response after first-line or salvage therapy. Multi-parametric medullary flow cytometry, FDG-PET/CT and DW- MRI were performed for each patient. DW-MRI identified more patients with residual focal bone lesions than FDG-PET/CT (21% vs. 6%, respectively). In contrast, residual focal bone lesions were only identified by FDG-PET/CT in five patients, suggesting that FDG-PET/CT and DW-MRI have additive prognostic power. Progression-free survival was significantly affected when residual focal bone lesions were detected, regardless of the imaging technique (DW-MRI or FDG-PET/CT). Prediction of outcome was significantly enhanced by the combination of intra-medullary MRD and functional imaging, resulting in double-negative and double-positive technical groups with very different outcomes. Patients identified as negative by both techniques had an excellent progression-free survival, and on the contrary, double-positive patients relapsed quickly.

Dynamic contrast-enhanced MRI (DCE-MRI) is a dynamic study of the accumulation and distribution of contrast medium (Gadolinium-DTPA) in the region of interest (in T1-weighted sequence) after a constant pump-controlled bolus injection. DCE-MRI-derived quantitative parameters such as amplitude A (correlated with blood volume) and exchange rate constant k_ep_ (reflecting vessel permeability and perfusion) reflect bone marrow angiogenesis/microcirculation, which plays a central role in the pathogenesis of MM, notably because it is correlated to MM-related bone disease and because of its pejorative prognostic significance [42,43,44]. In MM, preliminary studies have shown that the depth of the biochemical response after autologous stem cell transplantation is significantly correlated to decreased bone marrow microcirculation assessed by DCE-MRI-derived quantitative features [45,46].

## 5. Conclusions and Perspectives

FDG-PET/CT is the preferred imaging technique for the therapeutic assessment of MM. All large prospective studies of transplant-eligible newly diagnosed MM patient cohorts showed a strong and independent pejorative prognostic impact of not achieving complete metabolic response by FDG-PET/CT after therapy, especially before maintenance therapy. FDG-PET/CT and MRD (evaluated by multi-parametric flow cytometry or next generation sequencing at 10^−5^ and 10^−6^ levels respectively) results are complementary for MRD detection outside and inside the bone marrow. For patients with at least a biological complete response, achieving double negativity (FDG-PET/CT and intra-medullary MRD) is a predictive surrogate for patient outcome and a trustworthy and premature biomarker of treatment effectiveness.

However, FDG-PET/CT is not yet widely used in this setting in clinical practice. In order to extend its purpose, open issues need to be addressed, especially homogenization of interpretation guidelines for reporting results. Because the definition of complete metabolic response in FDG-PET/CT is not clearly established, the final results of prospective analyses currently underway, especially the CASSIOPET study, are expected to standardize the interpretation criteria of FDG-PET/CT after therapy to better define a cut-off to determine PET positivity/negativity for MRD assessment outside the bone marrow.

FDG-PET/CT is not suitable for MM therapeutic assessment and MRD detection in a sub-group of baseline symptomatic false-negative FDG-PET/CT patients. Novel PET tracers aiming to target different metabolic pathways or plasma cell receptors like CXCR4 are being employed in current studies and are expected to alleviate the known limitations of FDG. Whilst new sensitive functional MRI sequences such as DW-MRI are not routinely used in the clinic, its use in experimental research settings suggests that it will be very useful in complementing FDG-PET/CT imaging for MRD detection. DW-MRI needs to be further studied and correlated with clinical outcomes so simultaneous acquisition with a PET-MRI systemsounds attractive. PET-MRI will allow homogeneous prospective therapeutic assessment comparisons between FDG-PET/CT and DCE-MRI as well, particularly by comparing metabolic and bone marrow angiogenesis changes.

Finally, the influence of MRD assessment on treatment approaches still has to be defined.

## Figures and Tables

**Table 1:** Definition of complete metabolic response (including Fls and/or diffuse bone marrow involvement and/or EMD when present) according to main prospective studies assessing FDG-PET/CT for response to therapy in MM.

Study	Patients (N)	Definition of CMR
Bartel et al., 2009 [9]	239	100 % normalization of FDG uptake
Zamagni et al., 2011 [12]	192	Residual FDG uptake < 4.2
Usmani et al., 2013 [10]	302	100 % normalization of FDG uptake
Moreau et al., 2015 [14]	134	Residual FDG uptake ≤ hepatic background (DS 1-2-3)
Zamagni et al., 2018 [17]	236	Residual FDG uptake ≤ hepatic background (DS 1-2-3)
Moreau et al., 2019 [19]	268	Residual FDG uptake ≤ mediastinum background (DS 1-2) and uCMR when Residual FDG uptake ≤ hepatic background (DS 1-2-3)

CMR indicates complete metabolic response; Fls: focal bone lesions; EMD: extra-medullary disease; FDG-PET/CT: 18F-fluorodeoxyglucose-positron emission tomography with computed tomography; DS: Deauville scale; uCR: uncertain complete metabolic response.
